# Recombinant Poxvirus and the Tumor Microenvironment: Oncolysis, Immune Regulation and Immunization

**DOI:** 10.3390/biomedicines4030019

**Published:** 2016-08-12

**Authors:** Daniel W. Sharp, Edmund C. Lattime

**Affiliations:** 1 Rutgers Robert Wood Johnson Medical School, Rutgers, The State University of New Jersey, New Brunswick, NJ 08903-2681, USA; sharpdw@cinj.rutgers.edu; 2 Rutgers Cancer Institute of New Jersey, Department of Surgery, Rutgers Robert Wood Johnson Medical School, Rutgers, The State University of New Jersey, New Brunswick, NJ 08903-2681, USA

**Keywords:** oncolytic viruses, immunotherapy, GM-CSF (granulocyte-macrophage colony-stimulating factor), TRICOM (triad of costimulatory molecules), tumor microenvironment, poxvirus, vaccinia

## Abstract

Oncolytic viruses (OVs) are being extensively studied for their potential roles in the development of cancer therapy regimens. In addition to their direct lytic effects, OVs can initiate and drive systemic antitumor immunity indirectly via release of tumor antigen, as well as by encoding and delivering immunostimulatory molecules. This combination makes them an effective platform for the development of immunotherapeutic strategies beyond their primary lytic function. Engineering the viruses to also express tumor-associated antigens (TAAs) allows them to simultaneously serve as therapeutic vaccines, targeting and amplifying an immune response to TAAs. Our group and others have shown that vaccinating intratumorally with a poxvirus that encodes TAAs, in addition to immune stimulatory molecules, can modulate the tumor microenvironment, overcome immune inhibitory pathways, and drive both local and systemic tumor specific immune responses.

## 1. Introduction

Oncolytic viruses (OVs) are a family of viruses characterized by their preferential replication in—and subsequent lysis of—tumor cells following in vivo injection/trafficking to the site of the tumor. Early studies in animal models showed that OV treatment effectively inhibited tumor growth and prolonged survival [1,2]. Hypothesizing that active immune responses to the OVs would impede the success of therapy, some researchers tested adding immunosuppressive treatments [3]. However, it soon became apparent that OV therapy can also induce and enhance a systemic anti-tumor immune response, protecting treated mice from tumor re-challenge [4]. This created a rapidly growing field of OV-based cancer immunotherapy as researchers sought to harness the immune-enhancing capabilities of OVs. The advent of recombinant technology has allowed the development of OVs as vectors for gene therapy, encoding molecules to directly modulate immune responses. While there are a variety of different OVs actively being researched and developed for treatment, this review will focus primarily on the cancer therapeutic potential of poxviruses, especially vaccinia and fowlpox viruses, with recombinant expression of immunostimulatory molecules and tumor-associated antigens (TAAs) and in particular their ability to modulate the immune tumor microenvironment.

## 2. Poxviridae

### 2.1. Background

Poxviruses are large enveloped viruses with a double-stranded linear DNA genome ranging in size from 130 to 375 kbp and containing hundreds of genes [5]. Poxviruses replicate in the cytoplasm of infected cells, which allows them to serve as promising gene therapy vectors for transient transfection without fear of integration of foreign or recombinant DNA into the host genome. While not all members of the poxviridae family are oncolytic, they do show tropism for cancer, likely making use of cancer cells’ altered metabolism and downregulated innate immune responses [6]. While there are a number of genera and species of poxviruses (Table 1), the most extensively studied for human cancer therapeutics to date are members of the orthopoxvirus and avipoxvirus genera, and this review will primarily focus on them.

Orthopoxviruses are capable of infecting humans, the best known example being the variola virus, the causative agent of smallpox. Vaccinia virus (VV), another orthopoxvirus was first utilized by Edward Jenner in developing the smallpox vaccine, considered the first recorded instance of vaccination (hence the eponymous term, “vaccine”) [13]. When cancer virotherapy was first studied in the early 20th century, VV was tested by several groups as a treatment for metastatic melanoma [14]. Interestingly, early studies showed that intratumoral (i.t.) VV injection was able to induce tumor regression (evidence of its oncolytic capabilities) and even widespread vitiligo in some cases, likely due to the generation of a systemic immune response to antigens shared by the melanoma and melanocytes [15]. With the advent of modern molecular genetic techniques in the 1970s and 1980s, researchers characterized the genetics and protein expression of VV [16]. Work from the Moss lab in cloning, sequencing, and studying the roles of different genes and proteins led to the discovery that VV could serve as a recombinant vector for foreign genes [17]. In the decades since, researchers have shown that recombinant VV can be used as a vaccine against other viruses including hepatitis B [18], influenza [19], and even HIV [20,21].

These early successes and discoveries regarding VV led to its continued development and research for cancer treatment, and the isolation of several different VV strains. One particular strain of growing clinical interest is the Modified Vaccinia Ankara (MVA) strain. MVA was developed initially as an attenuated smallpox vaccine by passaging VV hundreds of times in chicken embryo fibroblasts, leading to the deletion and mutation of large sections of the genome [22]. These changes decreased the virulence of MVA, rendering it unable to replicate within mammalian cells. Despite this replication deficiency, MVA infects human hosts and induces immunity to variola, without the risk of serious side effects caused by VV infection, making it a useful vaccination vector [23]. It has also been shown in certain circumstances to be oncolytic, capable of inducing apoptosis in vitro [24].

The various VV strains developed for clinical use have pronounced cancer tropism due to deletions and mutations in various virulence genes, making VV well suited for OV therapy [25,26,27,28]. To date, a variety of recombinant VVs (rVVs) expressing immunostimulatory molecules and TAAs have been produced and tested in both preclinical and clinical settings [29,30,31].

While normally restricted to replication in birds, members of the avipoxvirus genus, specifically the canarypox virus (CV) and fowlpox virus (FV), have also been studied for human therapeutic applications. Because of their limited host range and inability to replicate in non-avian species, avipoxviruses are considered promising vectors for vaccination and gene therapy based on their maintained infectivity in mammals [32]. They began to be studied as cancer therapeutics after it was realized that when used as recombinant vaccines, VV therapy leads to high anti-viral immune responses that can limit the efficacy of repeated doses [33]. Since avipoxviruses to a large degree are non-cross-reactive with VV, they were theorized to be able to serve as part of a combination treatment strategy priming with VV and boosting with avipoxvirus [34]. While earlier studies focused on the use of CV, the use of FV has become more prevalent as research has showed it better able to encode transgenes and induce beneficial cytokines [35].

### 2.2. Mechanism of Action

**Figure 1:**
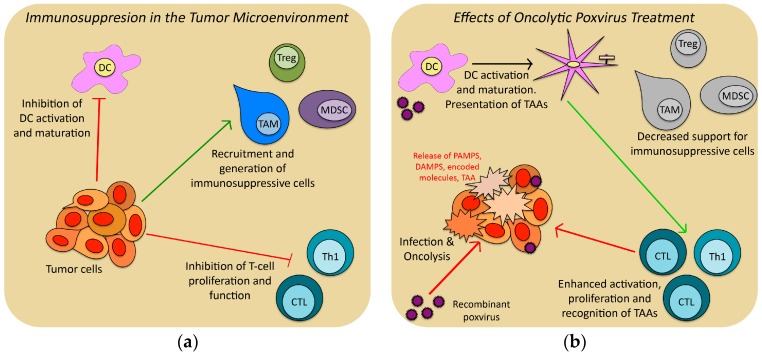
Schematic diagram showing how recombinant oncolytic viruses (OV) therapy can overcome the immunosuppressive effects of the Tumor Microenvironment (TME): (**a**) Tumor cell secretion of a variety of cytokines and other factors can inhibit productive immune response by dendritic cells (DC), cytolytic T-cells (CTL) and type-1 helper T-cells (TH1) and generate protective immunosuppressive cell populations such as tumor-associated macrophages (TAM), myeloid-derived suppressor cells (MDSC) and regulatory T-cells (Treg); (**b**) Intratumoral treatment with OV therapy can overcome the immunosuppressive TME by lysing tumor cells (releasing inflammatory signals), expressing immunostimulatory molecules within the TME (reversing the inhibition by tumor cells and removing the stimuli for immunosuppressive cells), and over-expressing tumor-associated-antigens (TAAs) for DC uptake and T-cell activation.

The mechanisms by which poxvirus can function as an OV therapy are multimodal, complex and still under investigation [36]. VV clearly exhibits tropism for cancer cells, preferentially infecting them and driving programmed cell death [37,38,39], but VV can also cause the death of cancer cells by indirect means. VV infection of a solid tumor causes the release of many pro-inflammatory signals that can lead to disruption of local vasculature [38] and the recruitment of both innate and adaptive immune cells [37,38]. It has been shown that the inflammation and recruitment of innate immune cells can indirectly kill tumor cells by blocking blood flow [40]. Of great interest to the field of cancer immunotherapy is the potential of VV to overcome the immune inhibitory effects of the tumor microenvironment (TME) and induce a systemic tumor specific immune response (Figure 1).

The TME is a term used to describe the local tissue area within and surrounding a tumor, including recruited immune cells, and surrounding stroma and vasculature. The TME has been extensively studied—especially with regards to how the tumor alters and controls its environment—and researchers have shown that cells and molecules within the TME can impact the local and by extension systemic immune response to tumor [41]. Tumors can secrete chemokines and cytokines to directly inhibit immune cell populations, including dendritic cells (DCs) and T-cells [42,43]. Tumors also recruit and activate immunosuppressive cell populations including T-regulatory cells (Tregs), myeloid derived suppressor cells (MDSCs), tumor-associated macrophages (TAMs) and tumor-associated neutrophils, which can protect the tumor from a functional anti-tumor immune response [44,45,46,47]. This immunosuppressive microenvironment can lead to immune ignorance—where antigen-presenting cells (APCs) are inhibited from taking up and presenting tumor antigens—and immune tolerance, where tumor-specific immune responses are blunted and downregulated. These immunosuppressive cells can thus impede the ability of OV immunotherapy to effectively break tolerance and induce anti-tumor immunity [48]. Any successful OV therapy will have to find ways to overcome the inhibitory effects of the TME.

One direct way that OVs may impact the immune microenvironment and immunization is via viral modulation of cytokine activity. Poxviruses have evolved defense and escape mechanisms to evade the host immune system that include the production of proteins that mimic the structure of cytokines and chemokines (virokines), and their receptors (viroceptors) [6,49,50,51]. Various poxviruses have been shown to express viroceptors for tumor necrosis factor [49], IL-1 beta [50], and IFN-gamma [52], and virokine homologues of epidermal growth factor [53] and IL-10 [54], and an inhibitor of granulocyte-macrophage colony-stimulating factor (GM-CSF) particularly relevant in this context [55]. In addition to prolonging viral infection, production of these factors within the TME is likely to disrupt the cytokine signaling produced by the tumor, hypothetically abrogating the immunosuppressive effects of the TME, allowing immune cells recruited by the viral infection to potentially target the tumor as well.

As mentioned previously, early trials with wild type VV in the 1960s showed signs of it being capable of inducing a systemic tumor-specific adaptive immune response [15]. This was likely accomplished by VV treatment serving as an in situ vaccination. By directly lysing tumor cells VV releases many different potential TAAs, overcoming immune ignorance. Uptake of antigens by APCs leads to the development of specific immune responses against a range of TAAs, a phenomenon termed epitope spreading. The overall inflammatory milieu produced by VV also provides co-stimulatory factors necessary to initiate both innate and adaptive anti-tumor responses. This adaptive response could then theoretically spread even to distant tumor sites untreated with VV.

In early OV therapy trials using wild-type VV, however, the in situ vaccination effect was modest at best (possibly due to the suppressive effects of the TME) and occurring only in a subset of patients [14,37]. These trials also found that VV therapy induced strong anti-viral immune responses, including the production of high titers of anti-VV antibodies, meaning prolonged VV therapy could potentially be impeded by active immune responses against VV [33]. Coincident with these studies, our group showed that even with high systemic anti-VV antibody titers, i.t. VV injection still led to productive local infection and expression of viral genes [37].

To maximize effectiveness of OV therapies and overcome immunosuppression within the TME, various strategies have been developed and tested. Taking advantage of the ability to encode OVs with immunostimulatory molecules and/or TAAs, we hypothesized that the best chance for success would be found in combining the viral lysis with pro-immune cytokines and, where possible, identified TAAs.

## 3. Recombinant Viruses

### 3.1. Virally Encoded Immune Stimulatory Molecules

The ability to encode genes for both cell surface and soluble immune modulating molecules and, when using poxvirus, the ability to encode multiple genes without compromising infectivity, makes poxvirus an ideal candidate for modulating the TME. The production of these virus-encoded immune-regulatory factors by the virus-infected cells within the TME would have the ability to enhance the anti-tumor immune response potentially primed by viral lysis. Potential immune-enhancing molecules tested include chemokines [56,57], regulators of differentiation and maturation [58,59], and enhancers of antibody-based [60], as well as cell-mediated [61] immunity and molecules with direct cytopathic effects on cancer cells [62]. To date, the most extensively studied poxvirus cancer gene therapies have used vectors encoding the cell surface costimulatory molecules B7-1 (CD80), ICAM-1 (CD54), and LFA-3 (human CD58; murine CD48) termed collectively as TRICOM and the soluble enhancer of antigen-presenting dendritic cells/pathways GM-CSF. Poxviruses encoding TRICOM have been shown to infect tumor cells as well as antigen presenting cells and enhance antigen specific T-cell responses [63]. Both recombinant FV (rFV) and rVV vectors expressing TRICOM were developed, and the 3 molecule TRICOM was shown to be more effective than treatment with vector encoding only one of its constituent costimulatory molecules [64]. Clinical trials of both VV-TRICOM and FV-TRICOM have shown them to be safe and capable of inducing T cell responses in cancer patients [65,66], and have been extensively studied clinically as part of combination therapies [67].

GM-CSF has a number of functions, primarily in the stimulation of the development of hematopoietic cells [68]. Of particular relevance here is its ability to activate antigen presentation by inducing the development of DCs from monocytes [69]. The proliferation of DCs is thought to be a primary mechanism by which GM-CSF has been shown to upregulate anti-tumor immunity. Our group and others have shown that GM-CSF can be stably expressed in rVV and rFV, and has the advantage of prolonged expression/production in infected compartments as opposed to injections of the soluble molecule which has a short local time of expression [37,69,70,71]. The inclusion of GM-CSF in anti-tumor treatments increases levels of both CD4+ and CD8+ T cells and can stimulate potent, long-lasting, and specific immunity [29,72].

Our group was the first to engineer vaccinia virus to encode human GM-CSF (VV-GMCSF) and to show that it could be safely used to treat melanoma patients [29]. In that 1999 trial, seven patients with surgically incurable cutaneous melanoma were treated with escalating i.t. doses of VV-GMCSF. Treatment led to tumor regression and dense immune infiltrate, including many CD4+ and CD8+ cells, in both treated and untreated lesions, some at distant sites which would be consistent with the generation of a tumor specific systemic immune response [29].

Building on the success of this study, the recombinant VV developed by our group has been licensed and continues to be developed under the name pexastimogene devacirepvec (Pexa-Vec; JX-594) [36,73]. Active clinical trials are underway using Pexa-Vec for treatment of hepatocellular carcinoma (in both adult and pediatric patient populations), colorectal cancer, neuroblastoma and Ewing sarcoma, among others [74,75,76,77]. In these trials, the virus is primarily administered intravenously instead of i.t., and is shown to traffic to and preferentially infect tumor, leading to prolonged infection primarily in tumor and not in other tissues [76]. Pexa-Vec is hypothesized to work by overcoming the inhibitory effects of the tumor microenvironment, and thus inducing long lasting T-cell mediated immunity even after the virus has been cleared [78]. Pexa-Vec is able to maintain antitumoral efficacy despite high neutralizing antibody titers [79], but inhibiting the effects of anti-VV neutralizing antibodies improved viral titers in blood and viral infection of tumor [80].

While not a poxvirus, another successful oncolytic DNA virus that should be mentioned is Talimogene Laherparepvec (T-VEC), a recombinant herpes simplex virus that also encodes GM-CSF [81]. T-VEC is the first oncolytic virus immunotherapy that has received FDA approval (for treatment of metastatic melanoma), and continues to be investigated in different applications [82]. T-VEC would be presumed to work similarly to Pexa-Vec by stimulating the development of a potent, tumor-specific immune response via direct oncolytic function complemented by production of recombinant GM-CSF [83].

While the use of GM-CSF encoding OV can lead to DC recruitment and overall immune activation, it should be noted that GM-CSF has also been shown to activate and recruit MDSCs, potentially countering its positive effects in stimulating adaptive immune responses [84,85]. GM-CSF also plays an important role in modulating TAMs in the TME, shifting the population towards the more beneficial M1 phenotype [86], which has been shown to improve cancer patient outcomes [87].

As a means of further enhancing the generation of systemic tumor antigen specific adaptive responses, we have taken the approach of combining genes for immune regulating molecules such as GM-CSF with encoding TAA.

### 3.2. Virally-Encoded Tumor-Associated Antigens and TME-Based Immunization

In early studies of immune regulation within the tumor microenvironment, we found that the immune regulating cytokine IL10 was overexpressed in biopsies of melanoma lesions in patients [88]. In dissecting the potential immune regulatory role of the TME IL10 expression we examined the effects of immunizing tumor bearing mice to VV encoding the *lacZ* gene with a readout of antibody response to the beta-galactosidase protein. These studies showed that immunizing within the IL10 high TME skewed the response to IgG1 (TH2) while immunizing in the contralateral flank resulted in a IgG2 (TH1) response [43] thus demonstrating that tumor-associated expression of immune modulatory molecules could have a dramatic effect on the nature of the resultant systemic response. These findings that the immunologic milieu at the tumor “immunizing” site coupled with our findings of an expansion of CD8+ tumor antigen specific CTL within the TME but not systemically [30] led us to focus on potential therapeutic approaches that combine recombinant OV therapy with vectors coexpressing TAA and immune regulatory molecules [30] while targeting the TME as the oncolytic “immunizing” site.

**Figure 2:**
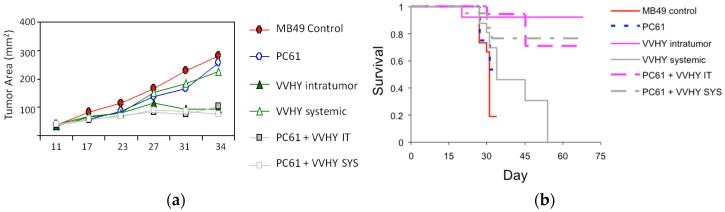
Neutralization of Tregs enhances the tumor growth-inhibiting effects of both systemic and intratumoral TAA vaccination. Combination treatment with the anti-CD25 monoclonal antibody PC61 and either systemic or intratumoral VV-HY (a cocktail of two rVVs encoding genes coding for two immunodominant tumor antigens) inhibits tumor growth (**a**) and results in prolonged survival (**b**) compared to treatment with either systemic or intratumoral VV-HY alone. Reprinted from [91], with permission from Elsevier.

While OVs encoding immunostimulatory molecules or TAAs have shown some clinical benefits, we hypothesized that combining them would prove more therapeutically effective. To investigate the role of the TME on OV therapy, our group conducted several studies using rVV constructs encoding GM-CSF and various TAAs. Utilizing our MB49 model, we showed that tumor-induced IL-10 production by immune cells in the TME inhibited the ability of DCs to induce an immune response [89]. Intratumoral (but not s.c.) treatment with a cocktail of rVVs encoding GM-CSF and relevant antigen-encoding genes resulted in a tumor specific systemic immune response, but was still unable to cause tumor regression [30]. Knowing that Tregs can produce IL-10 and inhibit immune responses in the TME [90], we made use of the Treg-depleting anti-CD-25 antibody PC61 to test if Treg ablation in our MB49 model would improve outcomes [91]. PC61 treatment alone had no effect on tumor growth, but PC61 synergized with our rVV treatment, improving the immune response and significantly slowing growth (Figure 2). Of note is how the addition of PC61 allowed s.c. rVV administration to achieve efficacy similar to i.t. rVV treatment, highlighting the impact that Treg inhibition in the TME had on our OV therapy.

**Figure 3:**
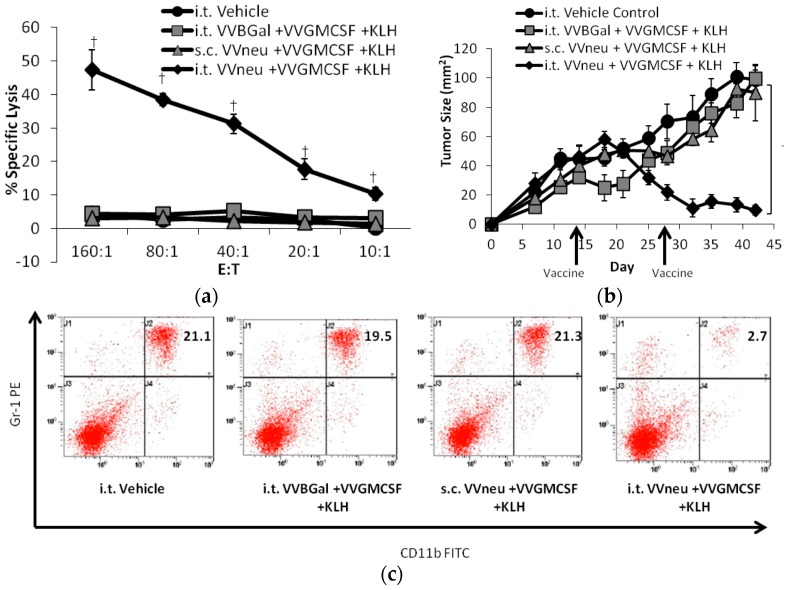
Vaccination into the tumor microenvironment with recombinant VV-neu leads to tumor regression, increased systemic CTL response, and reverses the systemic elevation in MDSC. (**a**) In a mouse HER2+ breast cancer mode, vaccination into the tumor microenvironment with recombinant vaccinia expressing the HER2/neu TAA (i.t. VV-neu + VV-GMCSF + KLH) results in a tumor specific CTL response; (**b**) regression of the primary tumor; (**c**) and a decrease in systemic MDSCs, whereas systemic treatment (s.c. VV-neu + VV-GMCSF + KHL) is not effective. Reprinted from [91], with permission from Elsevier.

We have found similar results using a second model (orthotopic growth of a syngeneic HER2/neu-overexpressing mammary carcinoma in FVB/N mice (NBT1) developed in our lab) where there was an expansion of MDSC in the TME. We found that i.t. injection of a combination of VV-GMCSF and VV-neu, but not VV-GMCSF alone, induced a systemic anti-neu CD8+ T-cell response (Figure 3a), led to tumor regression (Figure 3b), and decreased splenic and TME MDSC population (Figure 3c) [91,92]. It was noted that VV-GMCSF treatment in the absence of TAA actually led to a larger MDSC population in the TME as well as systemically. Similar to our MB49 findings, only i.t. rVV constructs that coexpressed GMCSF and the relevant TAA was effective in generating a systemic antitumor response, as s.c. injections showed no benefit. Together, these experiments by our group showcase how combination VV therapy, importantly coexpressing antigen, could disrupt the immune inhibition of the TME and drive systemic tumor immunity.

## 4. Conclusions

OV cancer therapy has progressed in the past decades, as recombinant technology enabled the deliberate expression of immunostimulatory molecules and TAAs. Prime-boost strategies combining different viral species avoid the downsides of anti-viral immune responses while maximizing anti-tumor responses, and injecting i.t. directly disrupts the inhibitory effects of the TME. Many clinical trials studying OVs have been conducted and are underway, with the first OV regimen gaining FDA approval last year. 

While OV therapy often is capable of great clinical benefit as a monotherapy, addition of chemotherapeutic agents may be of benefit. Several trials with various agents have shown synergistic potential [93,94,95,96]. Care in choosing agents that enhance instead of impede immune responses is essential, as is the determination of optimal route and timing of therapies. The recent trials showing clinical benefit of immune checkpoint inhibitors as single agents including antibodies against CTLA-4 and PD-1 or PD-L1 have opened up the potential for combinations with OV. Preclinical studies combining these agents have shown promising results, augmenting the immune responses, increasing survival, and in some cases leading to complete tumor regression [97,98,99].

Decades of research into poxvirus OV therapies have developed several successful clinical treatments and many more in preclinical studies. With the first OV therapy obtaining FDA approval, and increased interest in immunotherapy, the time has never been better for OVs. Poxviruses in particular, with their ability to express multiple recombinant molecules, disrupt the TME and overcome immune evasion, are excellent vectors for OV immunotherapy. Combining poxvirus OVs with next generation targeted agents and checkpoint inhibitors will likely lead to further successful treatments and improved patient outcomes in the future.

## Figures and Tables

**Table 1:** Example poxviruses.

Genera	Example Species	Primary Hosts	Human Infectivity	Use in Cancer Research and Therapy
Orthopoxvirus	Variola Vaccinia	Vertebrates and Arthropods	Yes	Extensive clinical trials
Avipoxvirus	Canarpox Fowlpox	Birds	Infects but does not replicate	Extensive clinical trials
Leporipoxvirus	Myxoma	Rabbits	Infects but does not replicate	Some preclinical models [7,8]
Yatapoxvirus	Tanapox	Monkeys and Baboons	Yes	Limited preclinical models [9,10]
Parapoxvirus	Orf	Sheep and Goats	Yes	Limited preclinical models [11,12]

## Abbreviations

The following abbreviations are used in this manuscript:

OV	oncolytic virus
TAA	tumor-associated antigen
TRICOM	triad of costimulatory molecules
GM-CSF	granulocyte-macrophage colony-stimulating factor
VV	vaccinia virus
MVA	modified vaccinia Ankara
FV	fowlpox virus
CV	canarypox virus
DC	dendritic cells
TME	tumor microenvironment
APC	antigen-presenting cell
Tregs	T-regulatory cells
MDSCs	myeloid derived suppressor cells
TAMs	tumor-associated macrophages
TANs	tumor-associated neutrophils
